# Evaluating the Usability of Electronic Patient-Reported Outcome Apps: Comment on a Symptom Management Platform for Outpatients With Advanced Cancer

**DOI:** 10.2196/42153

**Published:** 2023-08-07

**Authors:** Yu Haniuda, Michihiro Tsubaki, Yoshiyasu Ito

**Affiliations:** 1 Department of Emergency Nursing Kitasato University Hospital Kanagawa Japan; 2 School of Nursing Kitasato University Kanagawa Japan; 3 College of Nursing Art and Science University of Hyogo Hyogo Japan

**Keywords:** electronic patient-reported outcome, symptom management, advanced cancer, outpatient, follow-up, cancer

In the study “Implementing Symptom Management Follow-up Using an Electronic Patient-Reported Outcome Platform in Outpatients With Advanced Cancer: Longitudinal Single-Center Prospective Study,” Tang et al [1] suggest that using an electronic patient-reported outcome (ePRO) platform for following up on symptom management in patients with advanced cancer can improve completion rates and decrease the number of dropouts.

Tang et al [1] reported a high average compliance rate of 80.3% for all 8 out-of-hospital follow-up studies. Despite two reminders plus phone calls to patients who had not responded, 14 patients rejected participation in the follow-ups and 19 patients could not be contacted, resulting in about 20% of participants dropping out. According to a study by Lee et al [2], a decrease in engagement is related to the usefulness of an app for the continuous user. Chiu et al [3] also stated that user satisfaction is related to commitment. We believe that integrating these findings would lead to higher compliance in accepting the usability of an app and maintaining user satisfaction. Moreover, Lee et al [4] found that user satisfaction with utility is related to patient compliance with ePRO use. Therefore, user-friendly and attractive design is crucial for increasing satisfaction [5], especially for older adults. Apps should be equipped with not only new technologies but also interface designs that conform to the skills of older adults [5]. Therefore, we suggest evaluating the usefulness of an ePRO platform to determine user satisfaction, improve the user interface system, and reduce the dropout rate.

## Abbreviations

ePRO: electronic patient-reported outcome
